# Network Structure of Depressive Symptomatology in Elderly with Cognitive Impairment

**DOI:** 10.3390/medicina60050687

**Published:** 2024-04-23

**Authors:** Jeehyung Pyo, Hyukjun Lee, Jakyung Lee, Daseul Lee, Hyeona Yu, Shinn-Won Lim, Woojae Myung, Doh-Kwan Kim

**Affiliations:** 1 Samsung Medical Center, Department of Psychiatry, School of Medicine, Sungkyunkwan University, Seoul 06351, Republic of Korea; jocelyn79232@gmail.com; 2 Department of Neuropsychiatry, Seoul National University Bundang Hospital, Seongnam 13620, Republic of Korea; 82839@snubh.org (H.L.); jakyunglee@ewha.ac.kr (J.L.); daseul6866@naver.com (D.L.); hkhkh2142@naver.com (H.Y.); wmyung@snu.ac.kr (W.M.); 3 Department of Psychiatry, Seoul National University College of Medicine, Seoul 03080, Republic of Korea; 4 Department of Health Sciences and Technology, The Samsung Advanced Institute for Health Sciences & Technology Sungkyunkwan University, Seoul 06355, Republic of Korea; pimco8280@hanmail.net

**Keywords:** network analysis, depressive symptom, cognitive dysfunction, major depressive disorder, Alzheimer’s disease, mild cognitive impairment

## Abstract

*Objective and objectives*: Patients with cognitive disorders such as Alzheimer’s disease (AD) and mild cognitive impairment (MCI) frequently exhibit depressive symptoms. Depressive symptoms can be evaluated with various measures and questionnaires. The geriatric depression scale (GDS) is a scale that can be used to measure symptoms in geriatric age. Many questionnaires sum up symptom scales. However, core symptoms of depression in these patients and connections between these symptoms have not been fully explored yet. Thus, the objectives of this study were (1) to determine core symptoms of two cognitive disorders, Alzheimer’s disease and mild cognitive impairment, and (2) to investigate the network structure of depressive symptomatology in individuals with cognitive impairment in comparison with those with Alzheimer’s disease. *Materials and Methods*: This study encompassed 5354 patients with cognitive impairments such as Alzheimer’s disease (*n* 1889) and mild cognitive impairment (*n* = 3464). The geriatric depression scale, a self-administered questionnaire, was employed to assess depressive symptomatology. Using exploratory graph analysis (EGA), a network analysis was conducted, and the network structure was evaluated through regularized partial correlation models. To determine the centrality of depressive symptoms within each cohort, network parameters such as strength, betweenness, and closeness were examined. Additionally, to explore differences in the network structure between Alzheimer’s disease and mild cognitive impairment groups, a network comparison test was performed. *Results*: In the analysis of centrality indices, “worthlessness” was identified as the most central symptom in the geriatric depression scale among patients with Alzheimer’s disease, whereas “emptiness” was found to be the most central symptom in patients with mild cognitive impairment. Despite these differences in central symptoms, the comparative analysis showed no statistical difference in the overall network structure between Alzheimer’s disease and mild cognitive impairment groups. *Conclusions*: Findings of this study could contribute to a better understanding of the manifestation of depressive symptoms in patients with cognitive impairment. These results are expected to aid in identifying and prioritizing core symptoms in these patients. Further research should be conducted to explore potential interventions tailored to these core symptoms in patients with Alzheimer’s disease and mild cognitive impairment. Establishing core symptoms in those groups might have clinical importance in that appropriate treatment for neuropsychiatric symptoms in patients with cognitive impairment could help preclude progression to further impairment.

## 1. Introduction

Patients with cognitive impairment experience depression in all stages of dementia. It is known that depressive symptoms could be a prognostic feature of dementia development [1,2]. However, patients who are diagnosed with cognitive dysfunction often present similar symptoms to depression, such as loss of concentration, which might delay the diagnosis of depression [3,4]. For patients with cognitive impairment, their depressive symptoms are often misunderstood since symptoms of depression and cognitive impairment can overlap. Depressive symptoms and cognitive symptoms can be underdiagnosed. In addition, patients with dementia often have comorbidity of depression, different from patients who have no cognitive dysfunction [5]. One study has reported that 18% of individuals with cognitive dysfunction receive treatment for depression [6]. This presents the importance of early detection of depression and managing depressive symptoms in patients who have cognitive dysfunction.

Previous studies conducted on patients with cognitive impairment have found that neuropsychiatric symptoms such as depressive symptoms are related to progression to more severe types of cognitive impairment. Patients with cognitive impairment are accompanied by more neuropsychiatric symptoms than those without cognitive impairment. They are likely to progress to severe dementia. A previous study has also shown that patients with more neuropsychiatric symptoms are likely to have lower functional status than those with fewer neuropsychiatric symptoms [7]. This might have clinical importance in that appropriate treatment for neuropsychiatric symptoms in patients with cognitive impairment could help preclude progression to further cognitive and neuropsychiatric impairment.

Depressive mood is characterized by symptoms such as loss of interest, feeling of boredom, insomnia, change in appetite, worthlessness, and decreased energy. Many questionnaires measure depressive symptoms by adding up scales of depressive symptoms even if depressive symptoms are present in varying degrees [8]. Since psychiatric symptoms often coincide with many psychiatric disorders rather than specific symptoms of a specific disorder, it is useful to see symptoms as a network rather than a separate entity. For example, depressed mood often coincides with insomnia and loss of concentration. Those symptoms are not only commonly found in depressive disorder but also frequently found in anxiety disorders. Conventionally, depressive symptoms are scaled by summing up presence of each symptom, which is an easy method of estimating depressive symptoms. However, it is difficult to measure the dynamics between symptoms and variability. Symptom–symptom interaction can be explained by a network, which is useful for finding central symptoms with strong connections to other symptoms and finding bridging symptoms that connect one psychiatric disorder to other psychiatric disorders [9,10]. For example, a previous study on the network structure of anxious and depressive Chinese nursing students has found that irritability, depressed mood, worry, and trouble relaxing are central symptoms in the network, while depressed mood, nervousness, and anhedonia are bridge symptoms in the network. Central symptoms and bridge symptoms are useful for applying appropriate treatment and alleviating overall levels of anxiousness and depressed mood [11]. Therefore, network analysis of psychiatric symptoms such as many components of depressed mood can find dynamic relationships between interwoven symptoms of depression. Each symptom is noted as a node. Symptoms that are activated together are connected by edges. However, symptoms that are not activated together are not connected. If a patient shows a certain symptom, connected symptoms are likely to be more active than other symptoms. Insomnia is more connected to fatigue than other symptoms such as guilty feeling in the network analysis. Network analysis is also useful for identifying central symptoms of depression. Since a person having central symptoms is highly likely to have other linked symptoms in the network analysis, finding central symptoms of depression is essential for identifying the core depressive symptoms to target for possible effective intervention [12]. Additionally, the accuracy of network estimation and stability of the network structure should be determined to validate the structure. A bootstrap method could be applied to assess the accuracy and the stability of the network structure. A correlation stability coefficient could be used to assess the stability, and a bootstrapped difference test for edge weights could be used to determine differences in network connections and centrality estimates by different variables [9]. Analyzing symptom dynamics of depressive symptoms by network structure, assuming early diagnosis of depression, could make it easier to manage both depression and cognitive impairment and treat them with appropriate interventions. Thus, the objectives of the present study were (1) to determine core symptoms of two cognitive disorders, Alzheimer’s disease and mild cognitive impairment, and (2) to investigate the network structure of depressive symptomatology in individuals with cognitive impairment in comparison with those with Alzheimer’s disease. 

## 2. Methods and Materials

### 2.1. Study Sample

From 2005 to 2013, a total of 5354 participants were recruited and included in this research. Participants were outpatients in the clinical Research of Dementia of South Korea (CREDOS) who visited geriatric psychiatric clinics of university-affiliated hospitals to find associations between diagnosis of cognitive dysfunction and its risk factors. Clinicians selected patients who were diagnosed with either mild cognitive impairment or Alzheimer’s disease [8]. The diagnosis Alzheimer’s disease was established according to the National Institute of Neurological and Communicative Disorders and Stroke and the Alzheimer’s disease and Related Disorders Association criteria. The severity of the AD or disease stages were measured with the Clinical Dementia Rating as 1, 2, 3, meaning mild, moderate, or severe dementia, respectively. Examination was performed by a board-certified neuropsychologist. Patients were diagnosed as having mild cognitive impairment if they had subjective memory or other cognitive impairment reported by patients or informants or evident on neuropsychological tests or decreased function of instrumental activity of daily living (IADL). Their main caregivers had interviews with neuropsychologist and clinicians. Exclusion criteria were unstable psychiatric features (e.g., suicide attempts or psychiatric disorders such as schizophrenia or bipolar disorder), those who were unable to initiate an interview, and those who had severe illness such as diagnosis with severe medical conditions including malignancy and neurologic illnesses (e.g., epilepsy, encephalopathy, or encephalitis), and those with substance use disorders. All participants provided informed consent.

### 2.2. Depression Measurement

Depression was measured by the geriatric depression scale (GDS). The GDS is self-administered questionnaire. It has a score of 0 for no depression to 15 for severe depression. It uses a short form, different from its original 30-item questionnaire. GDS-15 is considered as effective as GDS-30 in measuring depression in elderly with high sensitivity and specificity [13,14]. This scale measures affective and cognitive function with responses being either 0 or 1. For five items (items 1, 5, 7, 11, and 13), a response of “no” indicates the presence of the depressive symptoms. However, for the other ten items, a response of “yes” indicates the presence of depressive symptoms. A score of greater than 5 indicates probable depression, while a score of greater than 10 implies that depression is highly likely [15].

### 2.3. Statistical Analysis

#### 2.3.1. Network Estimation

A network analysis was conducted using R4.3.2. The network analysis was performed with the Gaussian Graphical Model (GGM), an undirected network model which shows highly correlated variables using nodes and edges. Edges connect two nodes, showing their correlation. Thick edges represent higher correlations. Each node is a variable. Variables are connected by edges. Networks for each 15 GDS symptoms consisted of edges and nodes and were constructed using the R-package qgraph [16].

To prevent any noise and to show only strong relationships, the Graphical Least Absolute Shrinkage and Selection Operator (glasso) was implemented to shrink small edges to zero [17]. Strongly connected items of the GDS were identified as coherent subcommunities within the overall network through exploratory graph analysis (EGA) [18]. The Louvain algorithm is one of the most popular algorithms in determining modularity, the detection of communities. It applies an algorithm which finds a hierarchically structured modularity measure. The Louvain algorithm was applied to find optimal communities of the 15 items of the GDS [19]. The dimensional structure of the network and item stability within each community were calculated with an additional bootstrapping procedure using 2000 bootstrapped samples [9,20]. The bootstrapping method was applied to assess the accuracy of the network and assess centrality indices. It also determines whether there is any network centrality difference between variables.

#### 2.3.2. Network Centrality

Centrality measures the importance of nodes in a network [9]. Centrality of all variables is presented as strength, closeness, and betweenness centrality. Strength is measured by adding up all edge weights. Closeness was measured by adding up the shortest path lengths between nodes. Betweenness was measured by frequency of shortest path connecting nodes [11].

#### 2.3.3. Network Stability 

Robustness of centrality was assessed with R-package bootnet. Edge-weight accuracy was measured by 95% confidence intervals (CIs) from 2000 bootstrapped samples. The edge weight difference was analyzed to assess difference between the edge weight and strength. When the network has a stability measurement, it assesses whether the order of centrality indices is the same after repeating the estimation with fewer nodes [9].

Further analysis was conducted to determine whether there were differences between central symptoms between patients with mild cognitive impairment and those with Alzheimer’s disease. Group analysis was conducted by performing a bootstrapped difference test. Bootstrapping applies a repetition of network estimation [9].

#### 2.3.4. Network Comparison between Groups

A network comparison test was used for comparing the network between mild cognitive impairment and Alzheimer’s disease groups. The Network Comparison Test package was used to evaluate the network connectivity repeatedly. Differences in connectivity between the network, structure of the network, and all accumulated edges of the network were evaluated [21]. The network structure test used resampling permutation to compare data sets in terms of network structure, edge strength, and global strength [22].

## 3. Results

### 3.1. Participant Characteristics

Table 1 shows demographic characteristics of participants. Participants were 72.76 ± 8.47 years old in the group of patients diagnosed with Alzheimer’s disease. In the group of patients with Alzheimer’s disease, 31.29% of participants were male, and 68.71% were female. Participants were 69.73 ± 8.14 years old in the group of patients diagnosed with mild cognitive impairment. In the group of patients with mild cognitive impairment, 32.04% of participants were male, and 67.96% were female. In the Alzheimer’s disease group, 11.19% had higher than high school education. In the mild cognitive impairment group, 12.87% had higher than high school education. It was found that 87.82% in the Alzheimer’s disease group were employed, while 75.61% in the mild cognitive impairment group were employed. Regarding family history of Alzheimer’s disease, 80.31% had no family history in the Alzheimer’s disease group, and 81.60% had no family history of Alzheimer’s disease in the mild cognitive impairment group. Regarding alcohol use, 79.89% reported no alcohol use in the Alzheimer’s disease group while 76.53% reported no alcohol use in the mild cognitive impairment group. The numbers of depressive symptoms and GDS items for Alzheimer’s disease and mild cognitive impairment groups are presented in Table 2. Numbers of depressive symptoms present in mild cognitive impairment and Alzheimer’s disease groups were compared by the chi-square test, and the *p*-value was corrected by the Bonferroni method.

### 3.2. Network Construction for Mild Cognitive Impairment and Alzheimer’s Disease Groups

**Figure 1:**
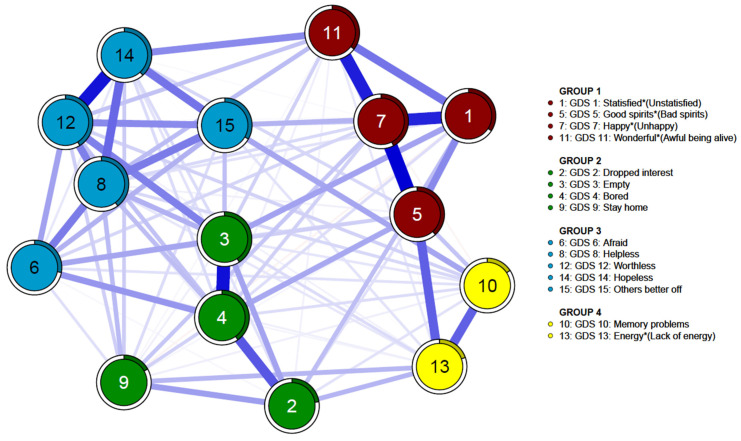
Network of GDS-15 items in patients with mild cognitive impairment (*n* = 3464). Each node is linked with edges, which represent the strength between nodes. * For five items (items 1, 5, 7, 11, and 13), a response of “no” indicates the presence of the depressive symptoms.

**Figure 2:**
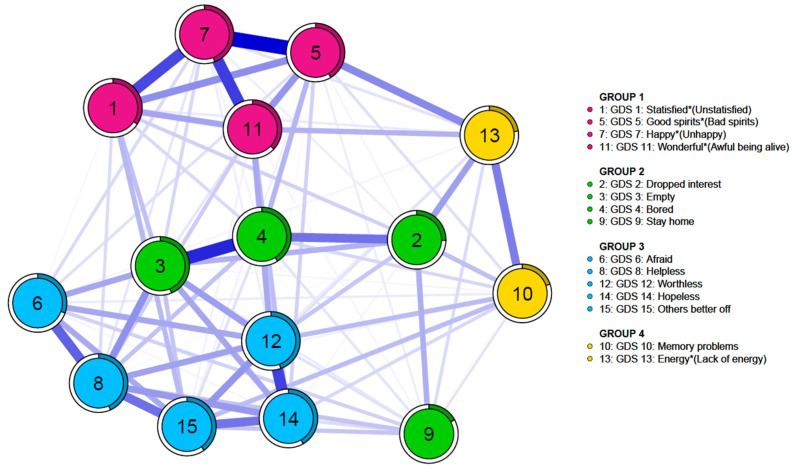
Network of GDS-15 items in patients with Alzheimer’s disease (*n* = 1889). Each node is linked with edges, which represent the strength between nodes. * For five items (items 1, 5, 7, 11, and 13), a response of “no” indicates the presence of the depressive symptoms.

The intercorrelation or network of geriatric depression symptoms in patients with mild cognitive impairment was estimated. Results are shown in Figure 1. Geriatric depression symptoms in patients with Alzheimer’s disease were estimated. Results are shown in Figure 2. Item communities in mild cognitive impairment and Alzheimer’s disease groups were detected using the EGA algorithm. They were categorized into four groups. Group 1 included item 1 “unsatisfied (reversed)”, item 5 “bad spirits(reversed)”, item 7 “unhappy (reversed)”, and item 11 “awful to be alive”. Group 2 included item 2 “dropped interest”, item 3 “empty”, item 4 “bored”, and item 9 “staying home”. Group 3 included item 6 “afraid”, item 8 “helpless”, item 12 “worthless”, item 14 “hopeless”, and item 15 “others better off”. Group 4 included items 13 “lack of energy (reversed)” and item 10 “memory problems”.

### 3.3. Centrality Indices and Edge Weights of Groups

**Figure 3:**
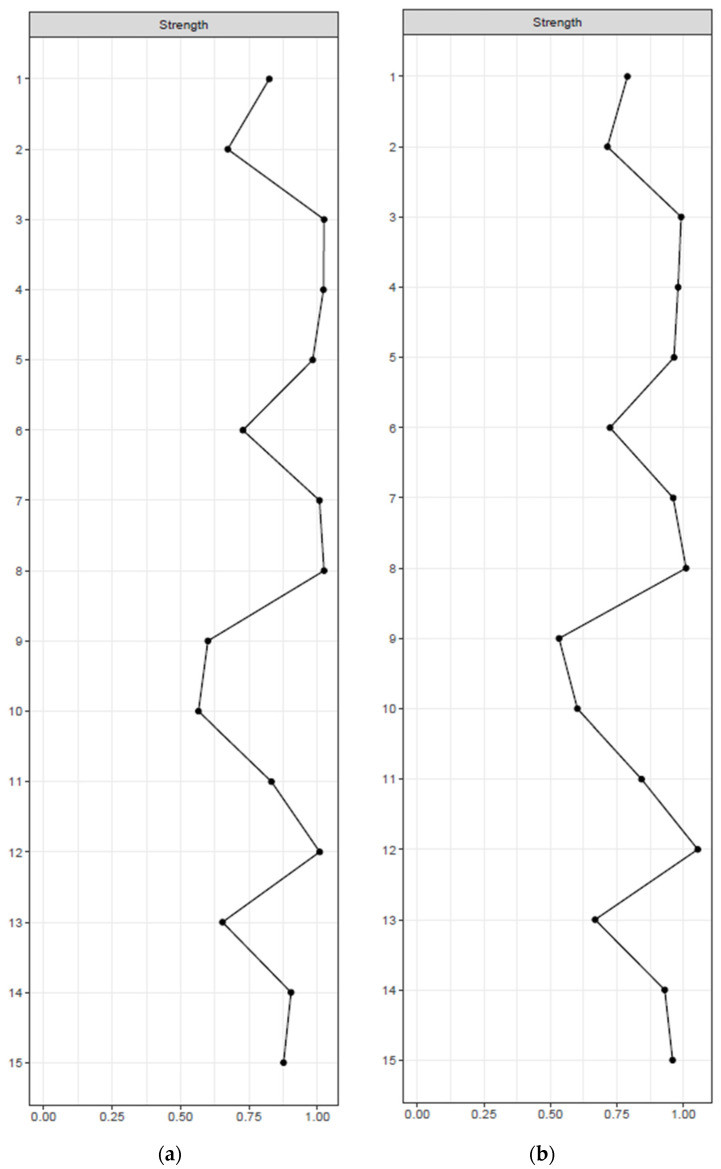
(**a**) Centrality indices of GDS-15 symptom items in patients with mild cognitive impairment. The *x*-axis represents standardized *z*-scores, and the *y*-axis represents symptom items. (**b**) Centrality indices of GDS-15 symptom items in patients with Alzheimer’s disease. The *x*-axis represents standardized *z*-scores, and the *y*-axis represents symptom items.

Centrality indices in mild cognitive impairment and Alzheimer’s disease are displayed in Figure 3a,b. The most central symptom in mild cognitive impairment was item 3 “empty”, with a strength of 1.024, a betweenness of 26, and a closeness of 0.005. Item 12 “worthless” was the most central symptom in Alzheimer’s disease, with a strength of 1.054, a betweenness of 22, and a closeness of 0.005. Bootstrapping results also showed corresponding centrality in mild cognitive impairment and Alzheimer’s disease groups (Figures S1 and S2). Bootstrapped edge-weight difference showed that item 5 “bad spirits” (reversed) and item 7 “unhappy” (reversed) had the strongest edges in mild cognitive impairment and Alzheimer’s disease groups (Figures S3 and S4).

#### The Highest Centrality Item in Mild Cognitive Impairment and Alzheimer’s Disease Groups

Centrality was analyzed in order to determine whether the centrality was consistent in both Alzheimer’s disease and mild cognitive impairment groups. Item 3 “empty” and item 8 “helpless” were the most central items in the group of mild cognitive impairment, while item 12 “worthless” and item 8 “helpless” were the most central items in the group of Alzheimer’s disease (Figures S1 and S2).

### 3.4. Network Stability in the Group of Mild Cognitive Impairment and Alzheimer’s Disease

We assessed the network stability indices of strength, betweenness, and closeness. For mild cognitive impairment, we found an excellent level of strength stability, indicating that 95% of the sample could be dropped while retaining a correlation of 0.7 along with the network structure invariant compared to the original structure. On the contrary, results of betweenness and closeness centrality showed an acceptable CS coefficient until 12.8% and 59.5% of samples were eliminated, respectively, suggesting that the betweenness and closeness might be less reliable indicators when the sample size is small.

For Alzheimer’s disease, we found excellent levels of strength and closeness stability. It was found that 95% of the sample could be dropped while retaining a correlation of 0.7 along with the network structure invariant compared to the original structure. On the contrary, results of betweenness centrality showed an acceptable CS coefficient until 28.3% of sample was eliminated, suggesting that betweenness might be a less reliable indicator when the sample size is small. Overlapping 95% CIs of edge weights are described in Figures S5 and S6. Results showed robustness of the network for mild cognitive impairment and Alzheimer’s disease groups. 

However, the edge between item 5 “bad spirits (reversed)” and item 7 “unhappy (reversed)” was the strongest edge. The edge between item 3 “empty” and item 4 “bored” was the second-strongest edge. The third-strongest edge was the edge between item 12 “worthless” and item 14 “hopeless”. The fourth-strongest edge was the edge between item 7 “unhappy (reversed)” and item 11 “awful to be alive” (reversed). The fifth-strongest edge was the edge between item 1 “unsatisfied (reversed)” and item 7 “unhappy (reversed)”. This was supported by the edge weight bootstrapped difference (Figures S3 and S4). For the mild cognitive impairment group, these five edges were significantly stronger than any other edges. However, these five edges were not statistically different from each other.

### 3.5. Network Comparison Test between Groups

The network comparison test was used to determine network structures and edge weights in mild cognitive impairment and Alzheimer’s disease groups. There was no statistically significant difference in network structure between the two groups (mild cognitive impairment vs. Alzheimer’s disease: M = 0.067, *p* = 0.811). Global strength was also compared between subgroups. There was no statistically significant difference between mild cognitive impairment and Alzheimer’s disease groups (S = 0.002, *p* = 0.993). 

## 4. Discussion

In this analysis, the main purpose was to find central symptoms in patients with cognitive decline, specifically in Alzheimer’s disease and mild cognitive impairment groups of patients. These two groups were compared to determine their differences. Item 12 “worthless” was the most central depressive symptom of the GDS. It measures depressive symptoms in old ages for patients with Alzheimer’s disease. For patients with mild cognitive impairment, item 3 “empty” was the most central symptom. The robustness of the result was validated through a bootstrapped difference test, which tested edge weights and centrality indices. The significance of results was determined based on the confidence interval.

In accordance with a previous longitudinal research study, our study sheds light on the relationship between depression dimensions and cognitive decline. In the previous research, these depression dimensions were categorized as dysphoric mood, withdrawal–apathy–vigor (WAV), anxiety, hopelessness, and subjective memory complaint based on the GDS, revealing significant insights. Specifically, dimensions like helplessness, worthlessness, and hopelessness were core elements of hopelessness. Interestingly, within the dysphoric mood category, the item “empty” was identified. However, patients with mild cognitive impairment experiencing “emptiness” did not exhibit significant long-term cognitive decline, in contrast to the “worthlessness” dimension, which showed a faster rate of cognitive decline [23]. Furthermore, another study involving 2000 individuals found a 37% increase in the risk of developing Alzheimer’s disease in the elderly who have the dimension of worthlessness of depressive symptoms [24]. Notably, item 12 “worthless” is not typically considered a primary symptom in the diagnosis of major depressive disorder. It is often presented as one of the depressive symptoms. However, our findings emphasize that patients with cognitive impairment who also experience feelings of worthlessness are of clinical significance and should be treated on par with other primary depressive symptoms within the geriatric depression network. The centrality of “worthlessness” in depressive symptom scales has been highlighted, surpassing decreased positive effect symptoms like insomnia, loss of energy, or loss of appetite. Bootstrap estimations demonstrated a strong correlation between “worthlessness” and self-blame components such as self-blame and hopelessness [25]. Moreover, previous research has categorized depressive symptoms in patients with cognitive impairment into two components: cognitive and vegetative symptoms [26]. Our study aligned with this categorization, as depressive symptoms such as “worthless” and “empty”, which fell within the cognitive depressive symptom category, exhibited high centrality within the network.

In this analysis, we grouped item 1 “unsatisfied (reversed)”, item 5 “bad spirits (reversed)”, item 7 “unhappy (reversed)”, and item 11 “awful being alive (reversed)” together in the community identification analysis. A previous research study has also combined these items and found that they share a common component known as “positive mental status” [8]. Additionally, the Beck Depression Inventory categorizes depression into two factors: cognitive/affective items (self-dislike, pessimism, worthlessness) and somatic/vegetative symptoms (decreased energy, appetite, and sleep changes) [27]. These two-factor systems have been validated in self-reported mood assessments within the cognitively impaired adult group [26]. In our analysis, item communities were detected by the EGA algorithm and categorized into four groups. Group 1 included items 1, 5, 7, and 11. Group 2 included items 2, 3, 4, and 9. Group 3 included items 6, 8, 12, 14, and 15. Group 4 included items 13 and 10. We similarly grouped items 1, 5, 7, and 11 together as a “positive mental status” group. Additionally, we identified three other distinct groups based on highly correlated components: items 2, 3, 4, and 9 as a “lower energy level” group; items 6, 8, 12, 14, and 15 as a “cognitive/non-vegetative components” group; and items 10 and 13 as a “decreased concentration” group. This categorization by highly correlated components enhanced the reliability and applicability of investigating depressive symptoms in patients with cognitive impairment.

In our analysis, items 7 “unhappy (reversed), 12 “worthless”, 8 “helpless”, 4 “bored”, 3 “empty”, and 5 “bad spirit (reversed)” showed centrality with statistically significant strength compared with the degree of other symptoms in our entire sample, with item 7 showing the strongest centrality (Figures S1 and S2). In other studies of GDS network analysis to determine central symptoms in depressive older adults, similar results were obtained, with items “hopeless” “unhappy (reversed)”, “worthless”, “emptiness”, “bad spirits (reversed)”, “bored”, and “helpless” identified to have higher strength, which represented central symptoms in geriatric depression [14], while item “helpless” was the only item that did not match with results of our study. “Helpless” was not identified to have a higher strength in our research. Since most symptoms were identified in a similar manner, this might imply the universality of centrality of depressive symptoms.

In mild cognitive impairment and Alzheimer’s disease groups of patients, item 5 “bad spirits (reversed)” and item 7 “unhappy (reversed)” were connected the most strongly. These closely linked symptoms suggest a potential interplay in the manifestation of depressive symptoms in individuals with cognitive impairment. Furthermore, results from NCT revealed no significant difference in network structure or global strength between mild cognitive impairment and Alzheimer’s disease groups. This notable finding suggests that depressive symptoms in individuals experiencing cognitive impairment exhibit similar network patterns and connectivity regardless of whether the patients have mild cognitive impairment or Alzheimer’s disease.

This study has several limitations. First, since it was a cross-sectional study, it was hard to detect the causal relationship between depressive symptoms and cognitive dysfunction. Also, the GDS had a relatively low sensitivity, which indicated a high rate of false negatives. GDS-30 is known to have higher sensitivity. However, the variability of depressive symptoms in the geriatric population is not well indicated in the GDS form in part because it is a self-reporting survey [28,29]. Despite these limitations, our study had a large number of samples with great generalizability.

## 5. Conclusions

Our study enhanced our comprehension of how depressive symptoms could manifest in individuals with cognitive impairment. Since core symptoms of depression in these patients and the connections between these symptoms have not been fully explored yet, findings of this study are anticipated to assist in the identification and prioritization of core depressive symptoms for patients with cognitive disorders. Cognitive disorders were divided into mild cognitive impairment and Alzheimer’s disease groups in the present study with core symptoms of “empty” and “worthlessness”, respectively. The network structure was evaluated through regularized partial correlation models. To determine the centrality of depressive symptoms within each cohort, network parameters such as strength, betweenness, and closeness were examined. The network comparison test revealed that the network was presented in a similar structure regardless of whether a patient had mild cognitive impairment or Alzheimer’s disease. Finding and treating core symptoms could alleviate overall symptoms of depression in patients with cognitive impairment. To build upon this knowledge, future research should be conducted to develop targeted interventions tailored to addressing these core symptoms in patients with Alzheimer’s disease and mild cognitive impairment.

## Figures and Tables

**Table 1:** Clinical and demographic characteristics of participants (*n* = 5353).

Characteristics	Mean (SD), *n* or %	
	AD (*n* = 1889)	MCI (*n* = 3464)
Age (years)	72.76 (8.47)	69.73 (8.14)
Gender (%)		
Male	31.29	32.04
Female	68.71	67.96
Education (%)		
High school or below	88.81	87.13
Others	11.19	12.87
Employment status (%)		
Unemployed	12.18	24.39
Employed	87.82	75.61
Family history of AD (%)		
Yes	19.69	18.40
No	80.31	81.60
Alcohol use status (%)		
Former or current	20.11	23.47
Never	79.89	76.53
Severity of AD, N		
Mild	1329	
Moderate	399	
Severe	43	
Not defined	118	

Abbreviations: SD, standard deviation; MCI, mild cognitive impairment; AD, Alzheimer’s disease.

**Table 2:** Analysis of item scores of geriatric depression scale (GDS) between MCI and AD groups.

	Items	Presence of Depressive Symptoms in GDS N (%)		Bonferroni Corrected *p*-Value
		MCI (*n* = 3464)	AD (*n* = 1889)	
1	Are you basically satisfied with your life? ^+^	2329 (67.2)	1234 (65.3)	2.624
2	Have you dropped many of your activities and interests?	2318 (66.9)	1356 (71.8)	<0.001
3	Do you feel that your life is empty?	1505 (43.4)	821 (43.5)	16.000
4	Do you often get bored?	1646 (47.5)	964 (51.0)	0.224
5	Are you in good spirits most of the time? ^+^	1823 (52.6)	1104 (58.4)	<0.001
6	Are you afraid that something bad is going to happen to you?	1529 (44.1)	702 (37.2)	<0.001
7	Do you feel happy most of the time? ^+^	2308 (66.6)	1249 (66.1)	11.472
8	Do you often feel helpless?	1047 (30.2)	619 (32.8)	0.896
9	Do you prefer to stay at home, rather than going out and doing things?	1079 (31.1)	661 (35.0)	0.080
10	Do you feel that you have more problems with memory than most?	2255 (65.1)	1195 (63.3)	3.024
11	Do you think it is wonderful to be alive now? ^+^	2451 (70.8)	1239 (65.6)	<0.001
12	Do you feel worthless the way you are now?	1258 (36.3)	792 (41.9)	<0.001
13	Do you feel full of energy? ^+^	1049 (30.3)	717 (38.0)	<0.001
14	Do you feel that your situation is hopeless?	1129 (32.6)	775 (41.0)	<0.001
15	Do you think that most people are better off than you are?	1014 (29.3)	611 (32.3)	0.320
	Total score, average ± SD	7.13 ± 2.32	7.42 ± 2.49	<0.001

^+^ Reversed items; Abbreviations: SD, standard deviation; MCI, mild cognitive impairment; AD, Alzheimer’s disease.

## Data Availability

No new data were created or analyzed in this study. Data sharing is not applicable to this article.

## Supplementary Materials

The following supporting information can be downloaded at: https://www.mdpi.com/article/10.3390/medicina60050687/s1, Figure S1. Bootstrapped difference tests between nodes in the network of GDS-15 symptom items among patients with mild cognitive impairment. Figure S2. Bootstrapped difference tests between nodes in the network of GDS-15 symptom items among patients with Alzheimer’s dementia. Figure S3. Bootstrapped difference tests between edge-weights in the network of GDS-15 symptom items among patients with mild cognitive impairment. Figure S4. Bootstrapped difference tests between edge-weights in the network of GDS-15 symptom items among patients with Alzheimer’s dementia. Figure S5. Bootstrapped confidence intervals of all edge-weights in the network of GDS-15 symptom items among patients with mild cognitive impairment. Figure S6. Bootstrapped confidence intervals of all edge-weights in the network of GDS-15 symptom items among patients with Alzheimer’s dementia.

## References

[1] Modrego P.J., Ferrandez J. (2004). Depression in patients with mild cognitive impairment increases the risk of developing dementia of alzheimer type: A prospective cohort study. Arch. Neurol..

[2] Ownby R.L., Crocco E., Acevedo A., John V., Loewenstein D. (2006). Depression and risk for alzheimer disease: Systematic review, meta-analysis, and metaregression analysis. Arch. Gen. Psychiatry.

[3] Mullerthomsen T., Arlt S., Mann U., Maß R., Ganzer S. (2005). Detecting depression in alzheimer’s disease: Evaluation of four different scales. Arch. Clin. Neuropsychol..

[4] Purandare N., Burns A., Craig S., Faragher B., Scott K. (2001). Depressive symptoms in patients with alzheimer’s disease. Int. J. Geriatr. Psychiatry.

[5] Knapskog A.-B., Barca M.L., Engedal K. (2014). Prevalence of depression among memory clinic patients as measured by the cornell scale of depression in dementia. Aging Ment. Health.

[6] Watson L.C., Garrett J.M., Sloane P.D., Gruber-Baldini A.L., Zimmerman S. (2003). Gruber-Baldini and S. Zimmerman. Depression in assisted living: Results from a four-state study. Am. J. Geriatr. Psychiatry.

[7] Edwards E.R., Spira A.P., Barnes D.E., Yaffe K. (2009). Neuropsychiatric symptoms in mild cognitive impairment: Differences by subtype and progression to dementia. Int. J. Geriatr. Psychiatry.

[8] Imai H., Yamanaka G., Ishimoto Y., Kimura Y., Fukutomi E., Chen W.-L., Matsuoka S., Tanaka M., Sakamoto R., Wada T. (2014). Factor structures of a japanese version of the geriatric depression scale and its correlation with the quality of life and functional ability. Psychiatry Res..

[9] Epskamp S., Borsboom D., Fried E.I. (2018). Estimating psychological networks and their accuracy: A tutorial paper. Behav. Res. Methods.

[10] Jones P.J., Ma R., McNally R.J. (2021). Bridge centrality: A network approach to understanding comorbidity. Multivar. Behav. Res..

[11] Bai W., Xi H.-T., Zhu Q., Ji M., Zhang H., Yang B.X., Cai H., Liu R., Zhao Y.-J., Chen L. (2021). Network analysis of anxiety and depressive symptoms among nursing students during the COVID-19 pandemic. J. Affect. Disord..

[12] Borsboom D., Cramer A.O. (2013). Network analysis: An integrative approach to the structure of psychopathology. Annu. Rev. Clin. Psychol..

[13] Azulai A., Walsh C.A. (2015). Screening for geriatric depression in residential care facilities: A systematic narrative review. J. Gerontol. Soc. Work.

[14] Kim K.M., Kim D., Chung U.S., Lee J.J. (2021). Identification of central symptoms in depression of older adults with the geriatric depression scale using network analysis and item response theory. Psychiatry Investig..

[15] Smarr K.L., Keefer A.L. (2011). Measures of depression and depressive symptoms: Beck depression inventory-ii (bdi-ii), center for epidemiologic studies depression scale (ces-d), geriatric depression scale (gds), hospital anxiety and depression scale (hads), and patient health questionnaire-9 (phq-9). Arthritis Care Res..

[16] Epskamp S. (2020). Psychometric network models from time-series and panel data. Psychometrika.

[17] Fried E.I., Epskamp S., Nesse R.M., Tuerlinckx F., Borsboom D. (2016). What are ‘good’ depression symptoms? Comparing the centrality of dsm and non-dsm symptoms of depression in a network analysis. J. Affect. Disord..

[18] Watkins M.W., Dombrowski S.C., McGill R.J., Canivez G.L., Pritchard A.E., Jacobson L.A. (2023). Bootstrap exploratory graph analysis of the wisc-v with a clinical sample. J. Intell..

[19] Traag V.A., Waltman L., Van Eck N.J. (2019). From louvain to leiden: Guaranteeing well-connected communities. Sci. Rep..

[20] Borsboom D. (2017). A network theory of mental disorders. World Psychiatry.

[21] Peralta V., Gil-Berrozpe G.J., Sánchez-Torres A., Cuesta M.J. (2020). The network and dimensionality structure of affective psychoses: An exploratory graph analysis approach. J. Affect. Disord..

[22] van Borkulo C.D., van Bork R., Boschloo L., Kossakowski J.J., Tio P., Schoevers R.A., Borsboom D., Waldorp L.J. (2023). Comparing network structures on three aspects: A permutation test. Psychol. Methods.

[23] Soleimani L., Soleimani L., Beeri M.S., Beeri M.S., Grossman H., Grossman H., Sano M., Sano M., Zhu C.W., Zhu C.W. (2022). Specific depression dimensions are associated with a faster rate of cognitive decline in older adults. Alzheimers Dement..

[24] Håkansson K., Soininen H., Winblad B., Kivipelto M. (2015). Correction: Feelings of hopelessness in midlife and cognitive health in later life: A prospective population-based cohort study. PLoS ONE.

[25] Harrison P., Lawrence A.J., Wang S., Liu S., Xie G., Yang X., Zahn R. (2022). The psychopathology of worthlessness in depression. Front. Psychiatry.

[26] Carvalho J.O., Tan J.E., Springate B.A., Davis J.D. (2013). Self-reported depressive syndromes in mild cognitive impairment and mild Alzheimer’s disease. Int. Psychogeriatr..

[27] Steer R.A., Ball R., Ranieri W.F., Beck A.T. (1999). Dimensions of the beck depression inventory-ii in clinically depressed outpatients. J. Clin. Psychol..

[28] Kørner A., Lauritzen L., Abelskov K., Gulmann N., Brodersen A.M., Wedervang-Jensen T., Kjeldgaard K.M. (2006). The geriatric depression scale and the cornell scale for depression in dementia. A validity study. Nord. J. Psychiatry.

[29] Chang Y.-P., Edwards D.F., Lach H.W. (2011). The collateral source version of the geriatric depression scale: Evaluation of psychometric properties and discrepancy between collateral sources and patients with dementia in reporting depression. Int. Psychogeriatr..

