# Supplementary material for: Network Structure of Depressive Symptomatology in Elderly with Cognitive Impairment
Source: Medicina (Kaunas). 2024 Apr 23;60(5):687. doi: 10.3390/medicina60050687 (PMC11123453; doi:10.3390/medicina60050687)

Supplementary Material

Network Structure of Depressive Symptomatology in Elderly with Cognitive Impairment

[2] Supplementary Figures

Figure S1. Bootstrapped difference tests between nodes in the network of GDS-15 symptom items among patients with mild cognitive impairment.

Figure S2. Bootstrapped difference tests between nodes in the network of GDS-15 symptom items among patients with Alzheimer's dementia.

Figure S3. Bootstrapped difference tests between edge-weights in the network of GDS-15 symptom items among patients with mild cognitive impairment.

Figure S4. Bootstrapped difference tests between edge-weights in the network of GDS-15 symptom items among patients with Alzheimer's dementia.

Figure S5. Bootstrapped confidence intervals of all edge-weights in the network of GDS-15 symptom items among patients with mild cognitive impairment.

Figure S6. Bootstrapped confidence intervals of all edge-weights in the network of GDS-15 symptom items among patients with Alzheimer's dementia.

Figure S1. Bootstrapped difference tests between nodes in the network of GDS-15 symptom items among patients with mild cognitive impairment.

Note: Gray boxes indicate that nodes are not significantly different from each other, while black boxes indicate that nodes are significantly different (α < 0.05) from each other.

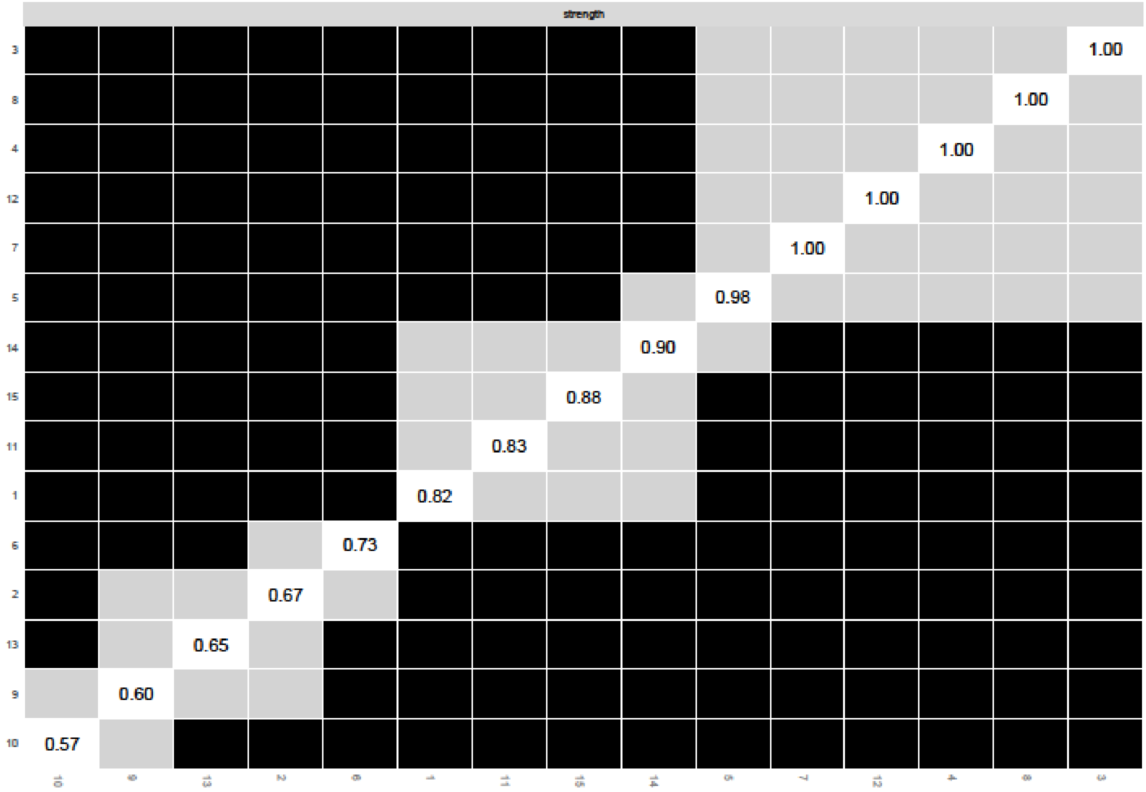

Figure S2. Bootstrapped difference tests between nodes in the network of GDS-15 symptom items among patients with Alzheimer's dementia.

Note: Gray boxes indicate that nodes are not significantly different from each other, while black boxes indicate that nodes are significantly different (α < 0.05) from each other.

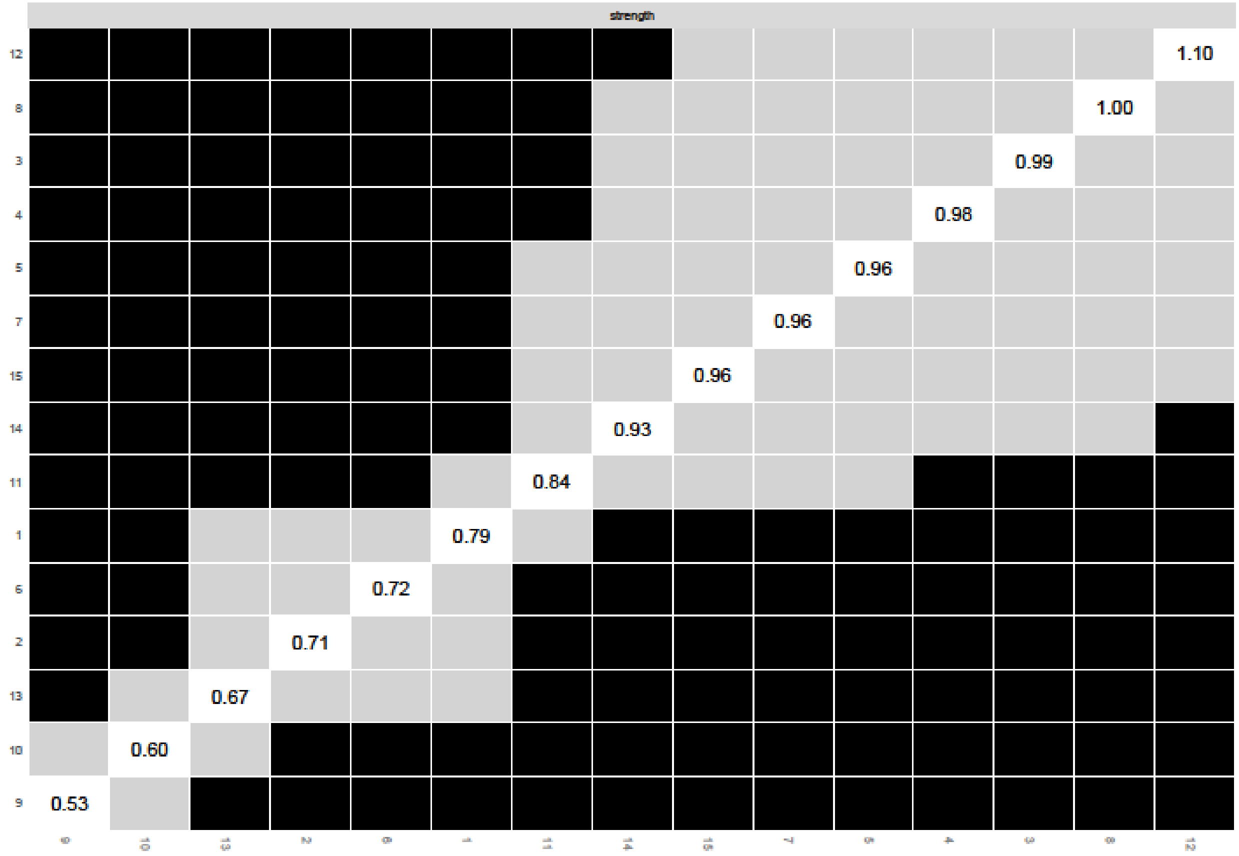

Figure S3. Bootstrapped difference tests between edge-weights in the network of GDS-15 symptom items among patients with mild cognitive impairment.

Note: Gray boxes indicate that nodes are not significantly different from each other, while black boxes indicate that nodes are significantly different (α < 0.05) from each other.

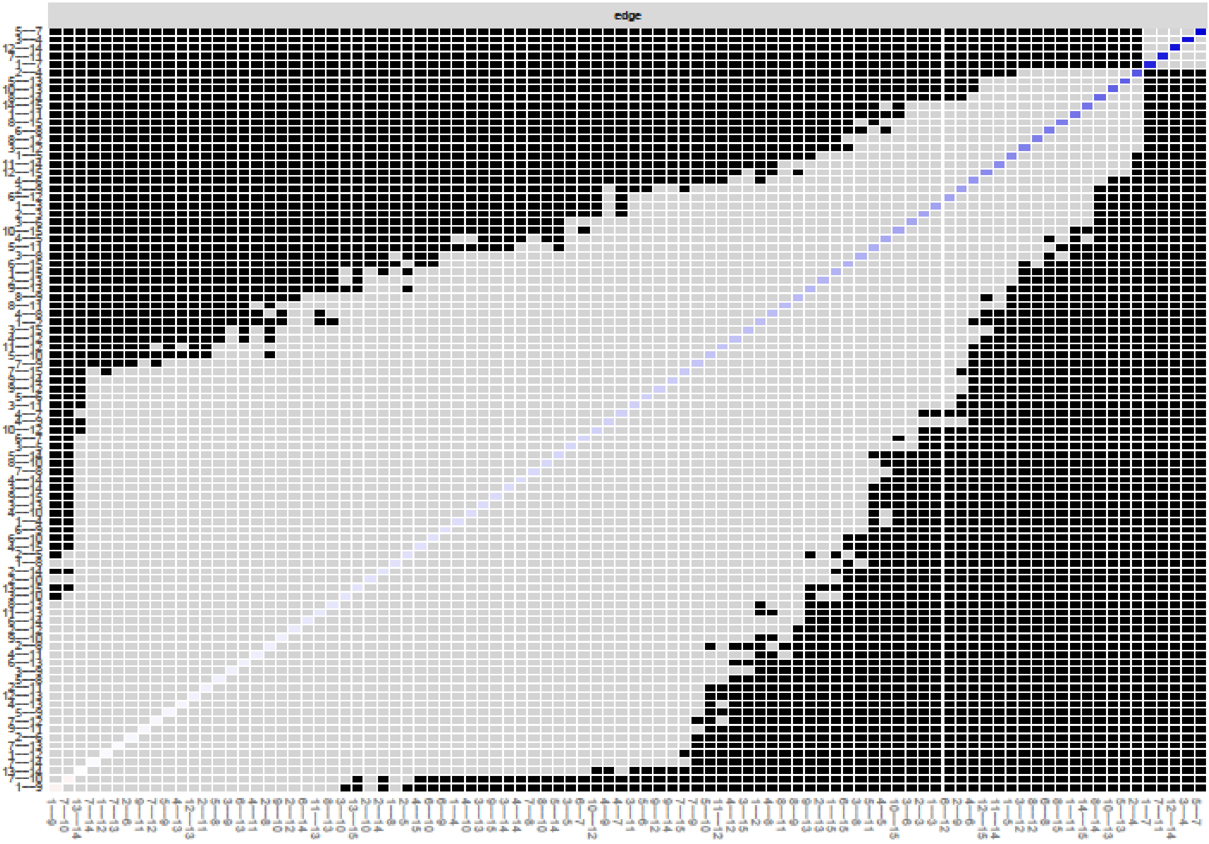

Figure S4. Bootstrapped difference tests between edge-weights in the network of GDS-15 symptom items among patients with Alzheimer's dementia.

Note: Gray boxes indicate that nodes are not significantly different from each other, while black boxes indicate that nodes are significantly different ($\alpha < 0.05$) from each other.

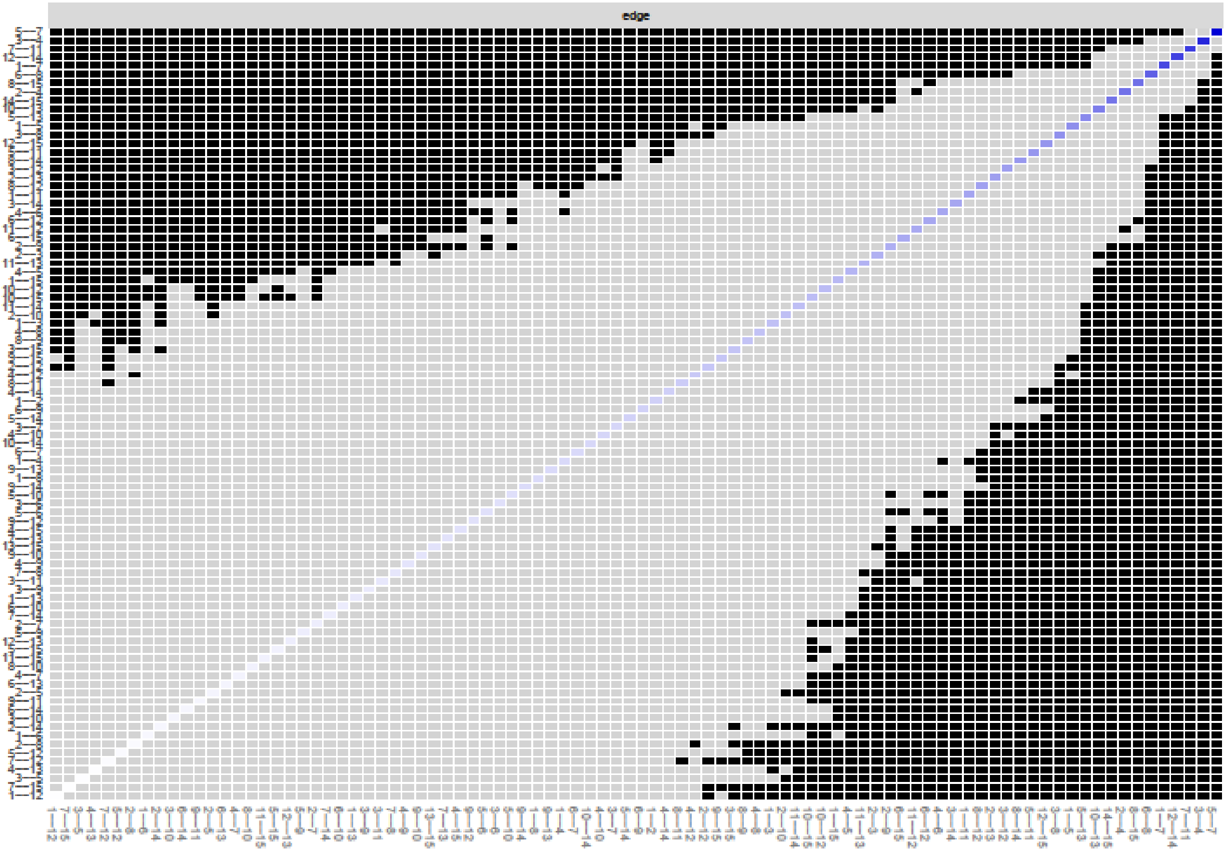

Figure S5. Bootstrapped confidence intervals of all edge-weights in the network of GDS-15 symptom items among patients with mild cognitive impairment.

Note: The red line indicates edge weights. The gray area indicates 95% confidence interval. Relatively narrow bootstrapped CIs indicate that estimates are precise.

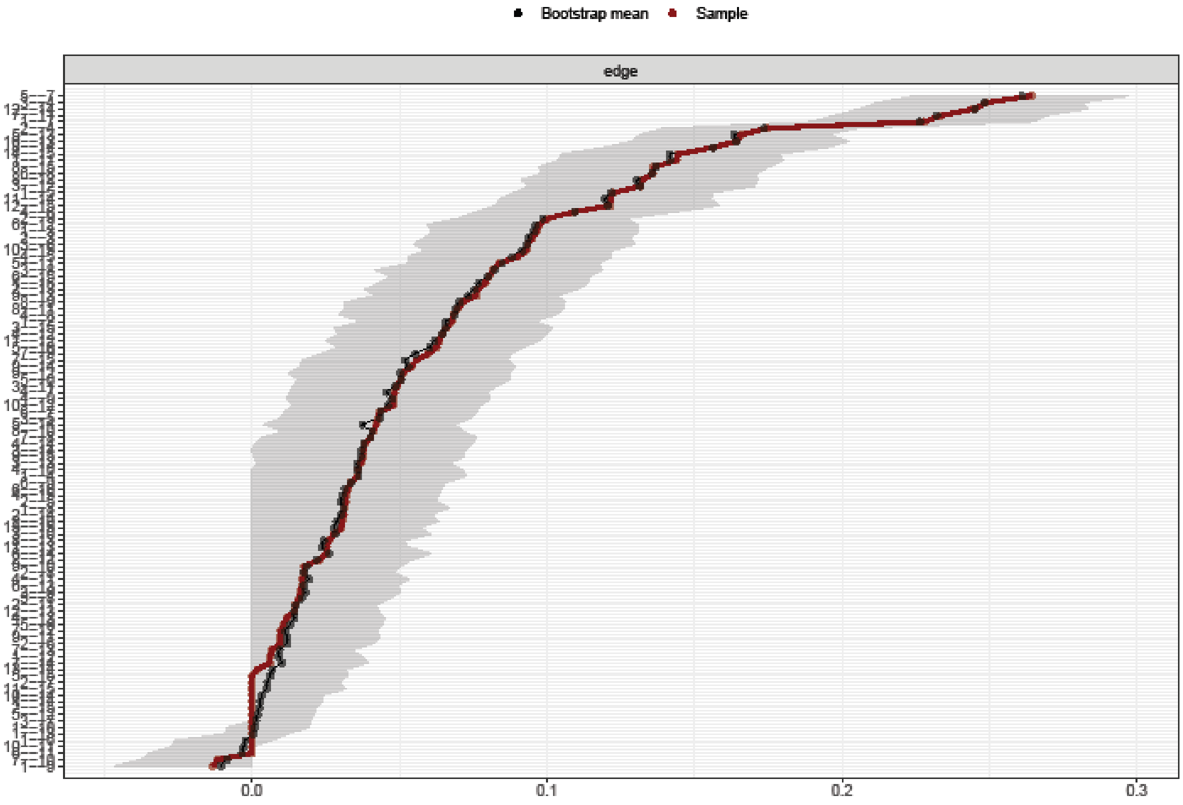

Figure S6. Bootstrapped confidence intervals of all edge-weights in the network of GDS-15 symptom items among patients with Alzheimer's dementia.

Note: The red line indicates edge weights. The gray area indicates 95% confidence interval. Relatively narrow bootstrapped CIs indicate that estimates are precise.

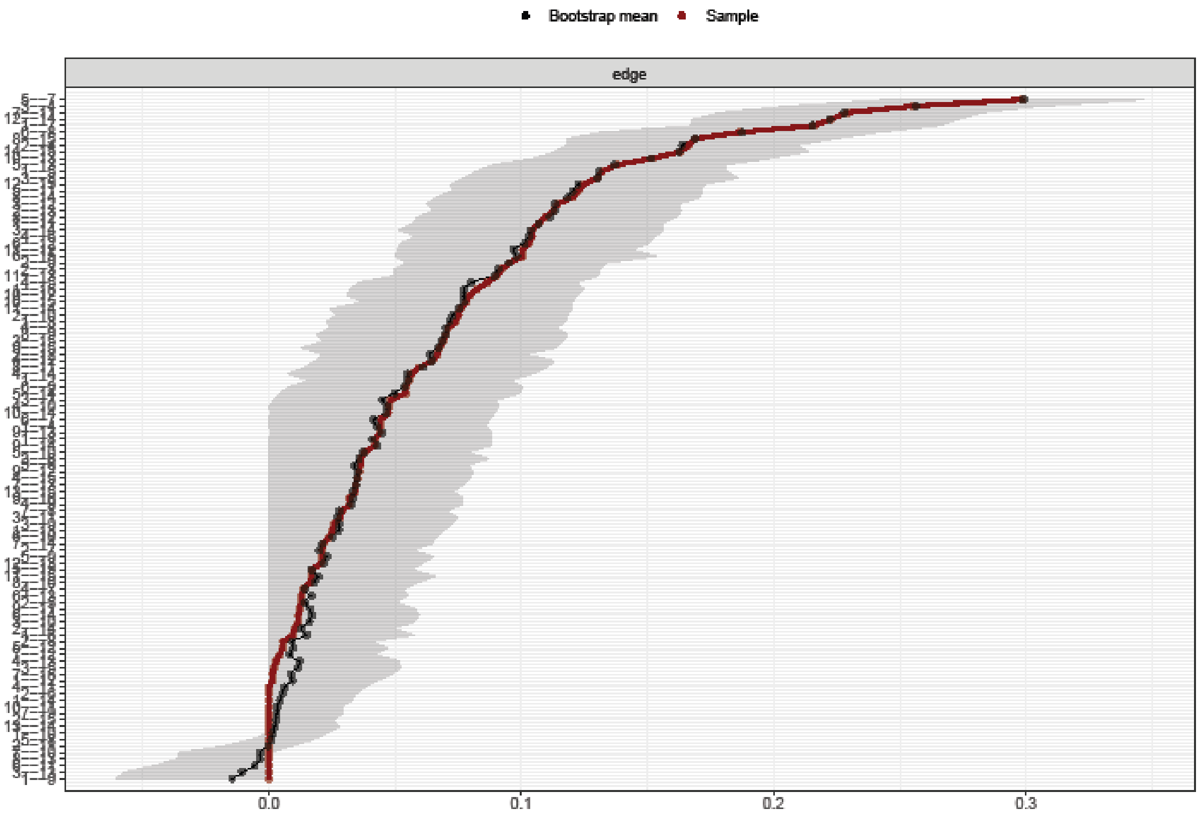

Supplement: Supplementary file 1 [file medicina-60-00687-s001.zip › medicina-2930669-supplementary.pdf]
